# Population Structure of and Conservation Strategies for Wild *Pyrus ussuriensis* Maxim. in China

**DOI:** 10.1371/journal.pone.0133686

**Published:** 2015-08-07

**Authors:** Tana Wuyun, Hitomi Amo, Jingshi Xu, Teng Ma, Chiyomi Uematsu, Hironori Katayama

**Affiliations:** 1 Paulownia Research and Development Center of China, Non-timber Forestry Research and Development of CAF, Weiwu Road, Zhengzhou City 450003, China; 2 Food Resources Education and Research Center, Faculty of Agriculture, Kobe University, Hyogo 675–2103, Japan; 3 Key Laboratory of Cultivation and Protection for Non-Wood Forest Trees and Non-wood Forest Products of SFA, Central South University of Forestry and Technology, Changsha, Hunan 410004, China; 4 Botanical Gardens, Graduate School of Science, Osaka City University, Osaka 576–0004, Japan; 5 Forestry technology Popularization Station of Beihai City, Beihai 536000, Guang Xi, China; Chiba University, JAPAN

## Abstract

*Pyrus ussriensis* Maxim. is native to the northern part of China, but whose habitats are currently being destroyed by environmental changes and human deforestation. An investigation of population structure and genetic diversity of wild Ussurian pear is a priority in order to acquire fundamental knowledge for conservation. A total of 153 individuals of wild Ussurian pear from the main habitats, Heilongjiang, Jilin, and Inner Mongolia in China, possessed low genetic diversity as a result of habitat fragmentation. The genetic diversity of the populations in Inner Mongolia and north east of Heilongjiang was especially low and there was the possibility of inbreeding. Wild Ussurian pears were divided into 5 groups based on the Bayesian clustering method using 20 nuclear SSRs (nSSRs) and 5 groups by haplotype distributions using 16 chloroplast SSRs (cpSSRs), and the populations in Inner Mongolia and north east of Heilongjiang represented unique genotypes. AMOVA indicated there was a 20.05% variation in nSSRs and a 44.40% variation in cpSSRs among populations. These values are relatively high when compared to those of other tree species. Haplotype E, positioned in the center of the cpSSR analysis network and showed the largest number of connections with other haplotypes, represented the most important haplotype. Inner Mongolia and the north east of Heilongjiang are two areas that need urgent conservation because of their genetic vulnerability and peculiarity. We determined 4 conservation units based on the clustering by nSSRs and cpSSRs, and geographic factor. This information is helpful in deciding the conservation strategies for wild Ussurian pear in China.

## Introduction

Investigating the genetic population structure, which includes genetic diversity, population differentiation and degree of gene flow [1], is very important in revealing the origin and the process of domestication. Furthermore, it is said that effective conservation strategies should be based on studies of genetic diversity [1]. On the basis of genetic information, concrete strategies, for example recovery of small and inbreeding populations, management of the fragmented populations, reducing introgression by hybridization with closely-related species, can be worked out [2].

Pears, which are cultivated throughout the temperate regions and have spread all over the world, belong to the genus *Pyrus* in the tribe Pyreae of subfamily Spiraeoidae in the Rosaceae [3]. The genus *Pyrus* is considered to have originated during the Tertiary period (65–55 million years ago) in the mountain regions of southwestern China [3]. China is one of three diversity centers for cultivated pear [4], thus there are various kinds of *Pyrus* species in China. Main cultivars in China are (1) Sand pear (*P*. *pyrifolia*), (2) Xingjiang pear (*P*. *sinkiangensis* Yu), (3) White pear (*P*. *bretschneideri* Rehd.), and (4) Ussurian pear (*P*. *ussuriensis* Maxim.). Ussurian pear is the most important cultivated pears in northern part of China. The differences between Ussurian pear and the other *Pyrus* species are as follows; (1) Morphological feature: short peduncle and pedicle, small fruit, the fruit shape is round or oblate, that skin color is yellow or brown, and the diameter is ranging from 2 to 6 cm with a 1 to 2 cm short pedicle [5]. The flower diameter is 3 to 3.5cm, a peduncle is 1 to 3cm, and 5 to 7 flowers in one flower cluster [6, 7]. (2) Cold resistance: this species can endure the temperature of -52°C. (3) After force-ripening, their eating quality can become better [5]. But classification of Chinese *P*. *ussuriensis* cultivars is problematic. Floristic studies [8] adopted the short fruit pedicel (<2 cm) as a discriminative character of the species from *P*. *pyrifolia* and *P*. *bretschneideri*. However, many cultivars with longer fruit pedicels have been classified as *P*. *ussuriensis* in the horticultural literature [5]. There are over 150 cultivars originated from this species in the northern east of China, but the domestication process haven’t revealed.

Cultivated Ussurian pears are considered to be derived from wild Ussurian pear native to the northern part of China, the east of Russia, the north of Korea and the north east of the main island in Japan [6, 7, 9]. In China, Heilongjiang, Jilin, and Inner Mongolia are considered the main distribution areas for wild Ussurian pears in the present time [10,11]. But now the forests in Heilongjiang and Jilin have been harmed by human development, so that the habitats of wild Ussurian pear are decreasing. In Inner Mongolia, there has been little rainfall for the past 30 years, which has led to serious drought damage, desertification and soil degeneration [11]. Consequently wild Ussurian pears in Inner Mongolia are now decreasing. According to research by Ma *et al*. [12] and Wuyun *et al*.[11], there were over 1,000 wild Ussurian pear trees in six natural habitats in 2009, but about two thirds of those trees have been killed by drought after a shortage of rainfall for more than 100 days in 2010 [11]. We need to understand the genetic population structure of these precious wild resources and conserve them before they become extinct.

In Japan, wild Ussurian pear trees and local varieties derived from *P*. *ussuriensis* var. *aromatica* (syn. to *P*. *ussuriensis* Maxim.) which is called ‘Iwateyamanashi’ are distributed mainly in the northern east part of the main island. The trees of Iwateyamanashi are usually found in local farmyards, on grazing land, along old roads, and occasionally on mountains [13, 14]. The phylogenetic relationships between *P*. *ussuriensis* var. *aromatica* and *P*. *ussuriensis* Maxim. in China is still unknown. The genetic investigation for wild *P*. *ussuriensis* Maxim. in China were especially deficient.

Currently, many molecular biological approaches using nuclear and chloroplast DNA markers have been applied to *Pyrus* to help understand the genetic diversity and population genetic structure [15, 16, 17, 18, 19, 11, 20]. These approaches have inspired the study of wild Ussurian pear in China. Cao *et al*. [21] surveyed the relationships between cultivated Chinese Ussurian pears and a few wild Ussurian pears, and Wuyun *et al*. [10] researched the genetic diversity of Chinese wild Ussurian pear using hypervariable regions of chloroplast DNA. But, to date, there has been no large-scale investigation of Chinese wild Ussurian pear using nuclear DNA.

Genetic analysis of population structure requires suitable markers. Microsatellite (Simple Sequence Repeat, SSR) has been used widely in construction of genetic maps, parentage and population genetic structure analysis [22]. Because SSR marker is PCR-based, highly reproducible, polymorphic, generally co-dominant and abundant throughout the eukaryotic genome, it has become a popular genetic marker in many species [23]. Recently many SSR primers were also developed for *Pyrus* [24, 25, 26, 27, 28] and *Malus* [29, 30, 31], and Yamamoto *et al*. [31] reported that SSR primers based on apple can also be transferred for *Pyrus*. Chloroplast DNA marker is a useful tool for evolutionary studies and population structure analysis because of the non-recombinant and uniparentally inherited nature of organelle genomes [32, 33]. Detecting useful polymorphisms at population levels is however often difficult because of the low level of substitutions in the chloroplast genome. The discovery of polymorphic mononucleotide repeats (cpSSR) in the chloroplast genome has revolutionized plant evolutionary biology and population genetics [34, 35]. The location of cpSSRs in *Pyrus* was reported in the entire sequence data of *P*. *pyrifolia* cpDNA [36]. The investigation of population genetic structure using these two kind of markers, can help to define conservation priorities for *Pyrus* [37].

The aim of this study is (1) to reveal genetic structure of wild Ussurian pear in China, such as genetic diversity, gene flow, and genetic divergence. This study is an initial step in determining suitable conservation units, and considering possible conservation strategies for the wild Ussurian pear. The second aim of this study is (2) to reveal the phylogenetic relationships between *P*. *ussuriensis* var. *aromatica* in Japan and *P*. *ussuriensis* Maxim., and get an insight into the origin of *P*. *ussuriensis*.

## Materials and Methods

### Collection sites and DNA extraction

**Fig 1:**
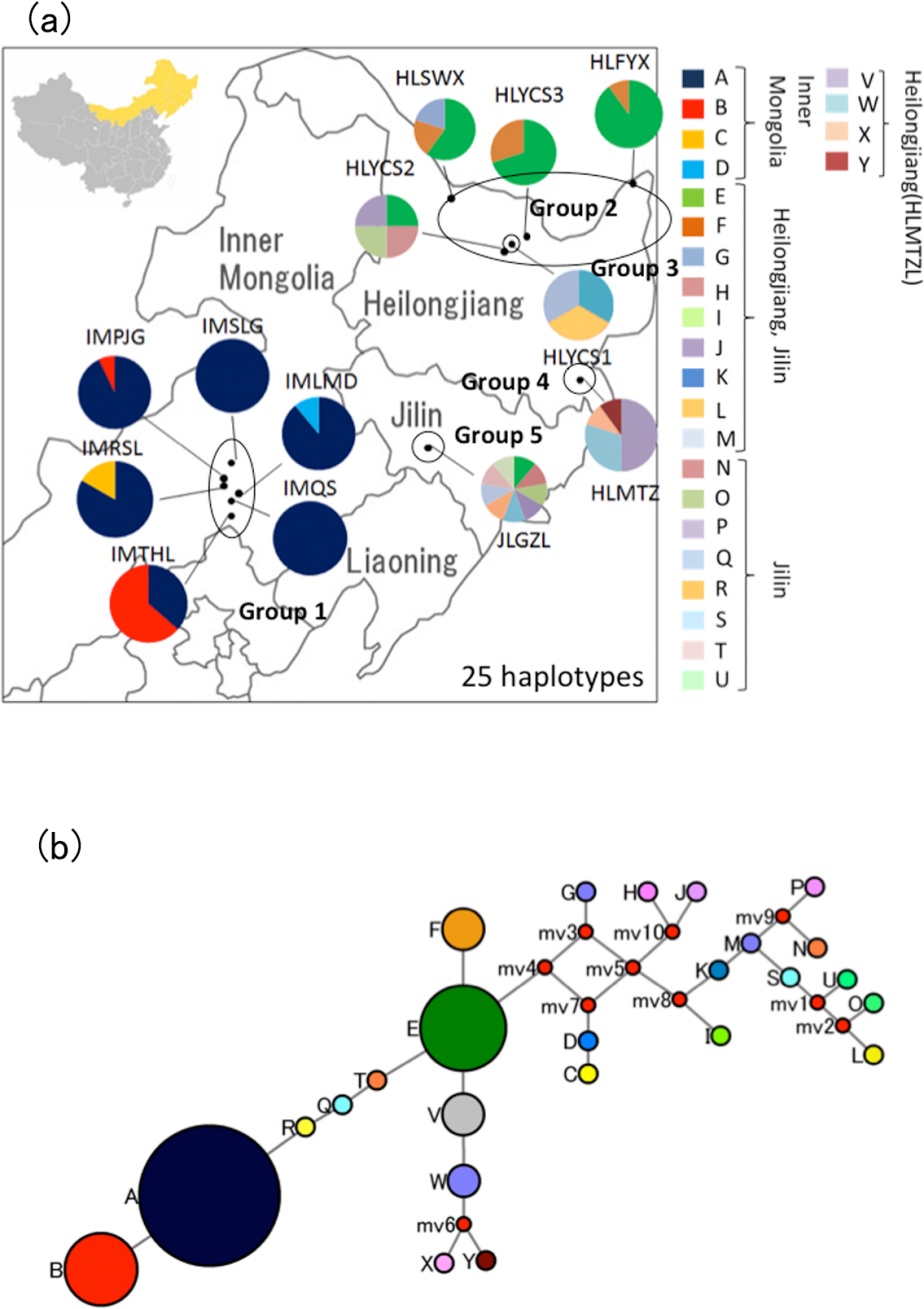
(a): Map of northeast China showing the distributions of chloroplast haplotypes (A~Y). (b): Median-joining network for 25 chloroplast SSR haplotypes and 124 individuals in Chinese wild Ussurian pears (Table 3). The haplotypes are indicated by circles and the colors correspond with the color of the haplotypes in Fig 1 (a), and small red circles show median vectors. The size of each pie chart is proportional to the frequency of corresponding haplotype. (1)-1 including 2 haplotypes was detected mostly in the north east of Heilongjiang, and (1)-2, (1)-3, and (1)-4 contained the haplotypes found in Jilin, Inner Mongolia, and Mudanzhang City, Heilongjiang, respectively. Most of the median vectors were included in (2).

Almost no investigations of wild Ussurian pears in China have been conducted until now, so their habitats were unclear. After explorations based on the old documentary records and interviews, 13 populations of Ussurian pears growing wildly were recognized. A total of 153 wild Ussurian pear (*Pyrus ussuriensis* Maxim.) individuals were collected from their main habitat, Inner Mongolia, Heilongjiang, and Jilin in China. We collected 13 populations comprised as follows: 91 individuals from 6 populations in Inner Mongolia, 70 individuals from 6 populations in Heilongjiang, and 12 individuals from a single population in Jilin (Fig 1). All of the samples were used for nSSRs analysis, and 145 individuals (86 individuals from 6 populations in Inner Mongolia, 49 individuals from 6 populations in Heilongjiang, and 10 individuals from a single population in Jilin) were used for cpSSRs analysis. Sampled locations were recorded as global positioning system (GPS) coordinates (Table 1). Classification of *Pyrus* based on those of Yu (1979) and Ohwi (1965). To avoid multiple sampling of closely related individuals such as half sib or full sib., a minimum distance of 10 m between individuals was maintained.

**Table 1:** Distribution of 13 wild populations of *P*. *ussuriensis* Maxim.

Population name	Location	Number of individual	Latitude (°N)	Longitude (°E)	Altitude (m)
IMQS	Qingshan forest farm, Keshiketengqi, Chifeng city, Inner Mongolia	18 (17)	43°14'61"–43°30'00"	117°49'96"–117°82'00"	950.50–1157.00
IMTHL	Tuohe forest farm, Keshiketengqi, Chifeng city, Inner Mongolia	26 (24)	43°04'00"–43°07'00"	117°52'89"–117–89'00"	1046.31–1157.00
IMPJG	Pijianggu, Keshiketengqi, Chifeng citiy, Inner Mongolia	20 (18)	43°36'00"–43°60'00"	117°46'53"–117°78'00"	1053.51–1106.00
IMRSL	Reshui forest farm, Keshiketengqi, Chifeng city, Inner Mongolia	6 (6)	43°36'16"–43°36'18"	117°46'74"–117°46'77"	1003.00–1124.00
IMLMD	Lamadong, Keshiketengqi, Chifeng city, Inner Mongolia	10 (10)	43°25'00"–43°42'00"	117°82'00"–117°83'00"	1108.21–1163.25
IMSLG	Shanligu, Keshiketengqi, Chifeng city, Inner Mongolia	11 (11)	43°38'01"–43°64'00"	117°46'46"–117°77'00"	1132.62–1271.00
HLYCS1	Wumahe, Youhao, Yichun, Heilongjiang	4 (4)	47°23'07"–47°59'01"	128°12'05"–128°48'01"	230.00–248.00
HLYCS2	Cuiluan, Yichun, Heilongjiang	9 (9)	47°23'07"–47°59'01"	128°12'05"–128°44'50"	300.00–500.00
HLYCS3	Meixi, Yichun, Heilongjiang	10 (10)	47°53'48"–47°53'86"	129°31'70"–129°31'94"	250.21–267.69
HLFYX	Fuyuan, Jiamusi, Heilongjiang	10 (10)	48°32'84"–48°32'85"	134°32'37"–134°32'44"	103.23–115.48
HLSWX	Sunwu, Heihe, Heilongjiang	7 (7)	49°03'20"–49°39'54"	127°40'00"–127°56'59"	120.00–400.00
HLMTZ	Muling, Mudanjiang, Heilongjiang	10 (9)	44°41'00"–44°41'40"	130°32'17"–130°28'17"	302.00–355.00
JLGZL	Gonhzhuling, Jilin	12 (10)	43°10'00"–44°09'05"	124°01'00"–125°18'00"	400.00–600.00

The numbers of individuals used for cpSSRs analysis is shown in parenthesis.

The Chinese cultivars used in this study as reference samples representative of cultivated pear gene pool were: 11 (10) Sand pear cultivars (*P*. *pyrifolia*: SL), 11 (9) Xingjiang pear cultivars (*P*. *sinkiangensis* Yu: XJL), 9 (7) White pear cultivars (*P*. *bretschneideri* Rehd.: BL), and 29 (16) Ussurian pear cultivars collected from 2 different places (*P*. *ussuriensis* Maxim.: QZL1 and QZL2). Furthermore, 12 (10) Japanese pear cultivars (*P*. *pyrifolia*: JAP), 15 (14) Japanese wild Ussurian pears (*P*. *ussuriensis* var. *aromatica* (Kikuchi *et* Nakai) Ohwi: Iwateyamanashi; IWT), and 11 (9) European pear cultivars (*P*. *communis* L.: EUR) were also used. The number shown in parentheses is the numbers of individuals used for cpSSRs analysis. No specific permissions were required for the described field studies: a) no specific permits were required for these locations/activities; b) locations were not protected; c) the field studies did not involve endangered or protected species. Young leaves were collected from the trees and DNA was extracted by A Plant Genomic DNA Kit (Tiangen Biotech, Beijing, China).

### Nuclear and chloroplast SSR markers

#### Development of cpSSR markers

Terakami *et al* [36] detected a total of 67 SSR regions (≧10 repeated motifs) by sequencing the entire chloroplast DNA of *Pyrus pyrifolia*. In this study, the software program, Primer3 (http://frodo.wi.mit.edu/cgi-bin/primer3/primer3_www.cgi), was utilized to design primer pairs flanking SSRs. The major parameters for primer design were as follows: primer length, about 20 bp; PCR product size, between 100 to 250 bp; optimum annealing temperature, 60°C; GC content, about 50%. Synthesized primer pairs (Life Technologies Japan Ltd) were tested to detect polymorphisms within 95 individuals of wild Ussurian pear endemic in Japan, and 16 cpSSR primers were selected for the present study (Table 2 and S1 Table).

**Table 2:** Genetic diversity within a population for 20 nSSR markers.

			nSSRs							
Type	Place	Population name	n	Na	Ne	Allelic richness	Ho	He	*FIS*	
Wild	China	IMQS	18	3.70	2.56	1.46	0.34	0.46	0.14	-
		IMTHL	26	3.90	2.49	1.48	0.31	0.48	0.32	**
		IMPJG	20	3.75	2.38	1.47	0.34	0.47	0.25	**
		IMRSL	6	2.90	2.16	1.45	0.29	0.45	0.26	-
		IMLMD	10	3.25	2.33	1.46	0.32	0.46	0.25	*
		IMSLG	11	3.05	2.11	1.46	0.33	0.46	0.13	-
		HLYCS1	4	2.45	2.04	1.46	0.39	0.46	-0.13	-
		HLYCS2	9	5.00	3.46	1.63	0.43	0.63	0.19	-
		HLYCS3	10	4.10	2.82	1.52	0.33	0.52	0.34	**
		HLFYX	10	2.85	1.99	1.39	0.30	0.39	0.36	**
		HLSWX	7	3.80	2.85	1.55	0.42	0.54	0.21	-
		HLMTZ	10	3.45	2.14	1.44	0.26	0.44	0.32	-
		JLGZL	12	5.50	3.49	1.61	0.44	0.61	0.06	-
Average of wild Ussurian pear in China			11.8	3.52	2.44	1.48	0.34	0.48	0.22	
Wild	Japan	IWT	15	5.85	3.67	1.71	0.56	0.71	0.16	**
Cultivars	China	QZL (*P*. *ussuriensis* Maxim.)	29	9.70	5.32	1.76				
		SL (*P*. *prifolia* Nakai)	11	6.05	3.91	1.72				
		XJL (*P*. *sinkiangensis* Yu)	11	5.60	4.04	1.73				
		BL (*P*. *bretschneideri* Rehd)	9	5.70	4.14	1.79				
	Europe	EUR (*P*. *communis* L)	11	5.30	3.44	1.73				
	Japan	JAP (*P*. *prifolia* Nakai)	12	4.20	2.63	1.67				
Average of cultivars			10.8	5.37	3.63	1.73				
Groups revealed by STRUCTURE analysis (Fig 4)	China	Group 1	91	5.00	2.63	2.92	0.36	0.54	0.26	**
		Group 2	20	4.75	2.74	3.18	0.37	0.57	0.32	**
		Group 3	20	6.10	3.49	3.79	0.47	0.66	0.25	**
		Group 4	12	5.50	3.49	3.93	0.47	0.64	0.17	**
		Group 5	10	3.45	2.14	2.89	0.35	0.59	0.26	**
Average of groups revealed by STRUCTURE analysis			30.6	4.96	2.90	3.34	0.40	0.60	0.25	

n: number of individuals analyzed in this study

na: average observed number of alleles

ne: effective number of alleles (Kimura & Crow 1964)

Ho: observed heterozygosity

He: expected heterozygosity

F_IS_: fixtation index

*P<0.05

**P<0.01,-non significant

h: haplotype diversity.

*PCR amplification*. The genotypes of a total of 273 individuals were determined using 20 nSSR markers (TsuENH155, NH029a, BGA35, NB104a, CH02e02, CH02b10, NB105a, NH009, NB141b, CH02d10b, CH03g06, NH039a, CH02b03, NH206a, NB109a, NH203a, EMPc114, CH04g12, CH02g01, and CH03d10) which were developed from a Japanese pear cultivar ‘Hosui’, an European pear cultivar ‘Bartlett’, and Apple (S1 Table and S2 Table). These 20 primers were selected separately from 17 linkage groups. For cpSSRs analysis, we analyzed 264 individuals using 16 cpSSR markers (Pchssr-3, Pchssr-6, Pchssr-14, Pchssr-17, Pchssr-19, Pchssr-27, Pchssr-31, Pchssr-36, Pchssr-39, Pchssr-42, Pchssr-44, Pchssr-45, Phssr-48, Pchssr-50, Pchssr-55, and Pchssr-60) which were developed from ‘Hosui’ (S1 Table). PCR reactions were carried out in a total volume of 10 μl containing 10 ng of genomic DNA, 2 μl of 5×PrimeSTAR Buffer, 0.8 μl of dNTP mixture, 0.5 units of PrimeSTAR DNA polymerase (Takara Bio, Japan), and 0.3 μM of each primer. Amplifications were performed in a My Cycler thermal cycler (BIO RAD, USA) under the following conditions: initial denaturation at 94°C for 2 min; followed by 10 cycles of 94°C for 1 min, 60°C for 1 min and 72°C for 2 min, the annealing temperature was reduced by 0.5°C per cycle; then followed by 30 cycles of 94°C for 1 min, 55°C for 1 min and 72°C for 2 min for 3 nSSR primers NB105a, NH009, and NB141b. Amplifications using the other nSSR and cpSSR primers were carried out in a S1000 Thermal Cycler (BIO RAD, USA) programed for an initial denaturation at 94°C for 3 min, followed by 30 cycles of 98°C for 10 sec, annealing at 55–60°C, and 72°C for 1 min. Amplified products were electrophoresed in 4.0% polyacrylamide gels and the banding patterns were visualized using the silver staining method described by Panaud *et al* [38] with modifications.

### Statistical data analysis

#### Genetic diversity in wild Ussurian pears

The genetic diversity as defined by nSSRs in each population was quantified in terms of the number of alleles per locus (Na), effective number of alleles (Ne), allelic richness (Ar), the mean observed heterozygosity (Ho), expected heterozygosity (He), and fixation index (*F*_*IS*_ = 1—Ho / He) using POPGENE 32 version 1.3.2 [39], FSTAT version 2.9.3.2 [40], Arlequin version 3.11 [41]. Deviations from Hardy-Weinberg expectations were determined by *F*_*IS*_ and their significance (*F*_*IS*_ ≠ 0) was examined using 1,000 permutation tests. For cpSSRs, the number of haplotypes per locus (Na), effective number of haplotypes (Ne) and Nei’s gene diversity were also calculated using POPGENE 32 version 1.3.2.

#### Genetic structure

The Bayesian model-based clustering programs STRUCTURE 2.3.4 [42] were employed to detect population structure and assign individuals to groups using the nSSRs under the admixture model and the option of correlated allele frequencies between populations. The cluster number (K) was set to vary from 1 to 20. The model was run as 10 independent simulations for each K and used a burn-in length of 100,000 and a run length of 1,000,000 MCMC iterations. The relationships between the K value and the data likelihood of K were plotted. To know the detail population structure of wild Ussurian pear in China, the run was conducted with only wild Ussurian pear in China. The cluster number (K) was set to vary from 1 to 10. The model was run as 10 independent simulations for each K and used a burn-in length of 100,000 and a run length of 1,000,000 MCMC iterations.

Analysis of molecular variance (AMOVA) was performed to examine the hierarchical genetic structure using the program Arlequin version 3.11 [43] for wild Ussurian pears. Genetic variance was partitioned into two levels using wild Ussurian pears, among populations and within a population. The significance of variance components and the differentiation statistics were tested with 1023 permutations.

To estimate the relative amounts of pollen and seed flow, we calculated the pollen to seed migration ratio (r = m_p_ / m_s_) as described by Mitsui *et al* (2010) using only wild Ussurian pears in China. The equation of m_p_ / m_s_ is based on the prediction that the levels of population differentiation can differ between nuclear and cytoplasmic markers because of their different modes of inheritance and that the extent of population differentiation is related to the relative levels of pollen and seed migration among populations [44, 45]. For biparentally inherited nuclear markers, gene flow occurs in both pollen and seeds, whereas the genes for maternally inherited cytoplasmic markers are only dispersed in seeds.

Mantel tests [46] were carried out using Arlequin to test the significance of isolation by distance patterns (IBD), regressing pairwise population *F*_*ST*_ / (1-*F*_*ST*_) values against the geographic distances between the respective pairs of populations [47]. The geographic distances used in this study were calculated using the Lambert-Andoye Method on the Internet; LatLng2Distance (http://lab.uribou.net/ll2dist/).

#### Phylogenetic tree

A dendrogram was constructed using nSSRs data to interpret relationships among populations by a neighbor-joining (NJ) method [48] based on Nei’s DA distance [49] by. Populations 1.2.32 [50]. Genetic distance was calculated in POPGENE 32 version.

A median-joining network including potential median vectors was constructed with wild Ussrian pears in China using Network 4.5.1.6 [51] using cpSSRs data. Any individuals with missing data were removed from this analysis (Table 3). We also calculated network including closely related species with wild Ussurian pears in China such as ‘cultivated Ussrian pears in China’ and ‘Iwateyamanashi’.

**Table 3:** Number and distribution of haplotype within 13 wild populations of *P*. *ussuriensis* Maxim.

Population name	Number of sample	haplotype																								
		A	B	C	D	E	F	G	H	I	J	K	L	M	N	O	P	Q	R	S	T	U	V	W	X	Y
IMQS	13	13																								
IMTHL	22	8	14																							
IMPJG	14	13	1																							
IMRSL	6	5		1																						
IMLMD	9	8			1																					
IMSLG	9	9																								
HLYCS1	3											1	1	1												
HLYCS2	4					1			1	1	1															
HLYCS3	10					7	3																			
HLFYX	10					9	1																			
HLSWX	5					3	1	1																		
HLMTZ	10																						5	3	1	1
JLGZL	9					1									1	1	1	1	1	1	1	1				

The individuals including missing data were removed.

#### Flower and fruit morphology

Five flower morphologies: (1) Flower diameter, (2) Petal length, (3) Petal width, (4) Petal length/ Petal width, and (5) Peduncle length were measured in 7 populations (IMQS, IMTHL, IMLMD, IMSLG, HLYCS3, HLFYX, and HLMTZ) and 26 Ussurian pear cultivars. We measured up to 10 randomly selected flowers from each individual (S7 Table). Six fruit morphologies: (1) Fruit diameter, (2) Fruit length, (3) Fruit width, (4) Fruit length/ Fruit width, (5) Peduncle length, and (6) Calyx existence were calculated in 5 populations (IMQS, IMTHL, IMPJG, IMSLG, and HLYCS3) and an Ussurian pear cultivar ‘Nanguoli. We measured 20 randomly selected fruits from each individual (S8 Table).

To determine the morphological features for each population, we performed a discriminant analysis, using the program JMP 8 (SAS Institute Inc., Cary NC, USA). Discriminant analysis was performed using all of the morphological traits except for calyx existence.

## Results

### Chloroplast haplotypes characterized by 16 cpSSR markers

A total of 105 chloroplast haplotypes were identified amongst the samples such as wild individuals and cultivars using 16 cpSSRs. These cpSSRs harbored enough genetic diversity to represent the genetic variation of the *Pyrus* species. Twenty five haplotypes were represented in wild Ussurian pears in China (Fig 1A, Table 3). Wild Ussurian pear in China were divided into 5 groups based on the haplotype distribution; (1) Inner Mongolia; IMQS, IMTHL, IMPJG, IMRSL, IMLMD, and IMSLG, (2) The north of Heilongjiang; HLSWX, HLYCS2, HLYCS3 and HLFYX, (3) HLYCS1: This population had unique haplotype and didn’t have the haplotype E which is representative haplotype in Heilongjiang, (4) HLMTZ and (5) JLGZL. Four haplotypes, A, B, C, and D, were found among the 6 Inner Mongolian populations. Seventy six percent of the 73 individuals from Inner Mongolia could be assigned to haplotype A, which was considered to be the major haplotype amongst 6 Inner Mongolian populations. Thirteen haplotypes, E, F, G, H, I, J, K, L, M, V, W, X, and Y were detected in the 6 populations from Heilongjiang, and 9 haplotypes, E, N, O, P, Q, R, S, T, and U were identified in the population from Jilin.

Haplotype E was detected in both the Heilongjiang and Jilin populations, and represented 41% of the 51 individuals from these populations. Seventy one percent of the 20 individuals represented by haplotype E were detected in the north east in 2 populations from Heilongjiang (HLYCS3 and HLFYX), therefore haplotype E was the most common haplotype for wild Ussurian pears in the north east of Heilongjiang (Fig 1A, Table 3).

The relationships between 25 cpSSR haplotypes detected in wild Ussurian pears in China were analyzed by a median-joining network model (Fig 1B). The haplotype network is an indication of the minimum number of evolutionary events separating each haplotype. The Network indicated that haplotype A is derived from E via T, Q, and R, and the other haplotypes also seemed to be subdivided from E. Some cultivated Ussurian pears in China also derived from haplotype E (S1 Fig). Consequently, haplotype E is considered to be the divergent center of wild Ussurian pears. Nine median vectors (mv) inferred from the network might have arisen from homoplasious mutation based on mononucleotide repeats in cpSSRs (Fig 1B). Possible shortest least complex phylogenetic tree (maximum parsimony tree) was also reconstructed by using the Network software, and the result was same to that with median-joining method.

### Genetic structure in Chinese wild Ussurian pears

The results of the AMOVA for nSSR showed that 20.05% of the variation was due to differences among populations, and 79.95% within each population. For cpSSR, 44.40% of the total genetic variation was due to differences among populations, and 55.60% within each population. Differentiation among populations for cpSSRs was more than twice as large as that of nSSRs. The calculated ratio of pollen flow to seed flow were 5.8: 1 in 13 wild populations from China.

**Fig 2:**
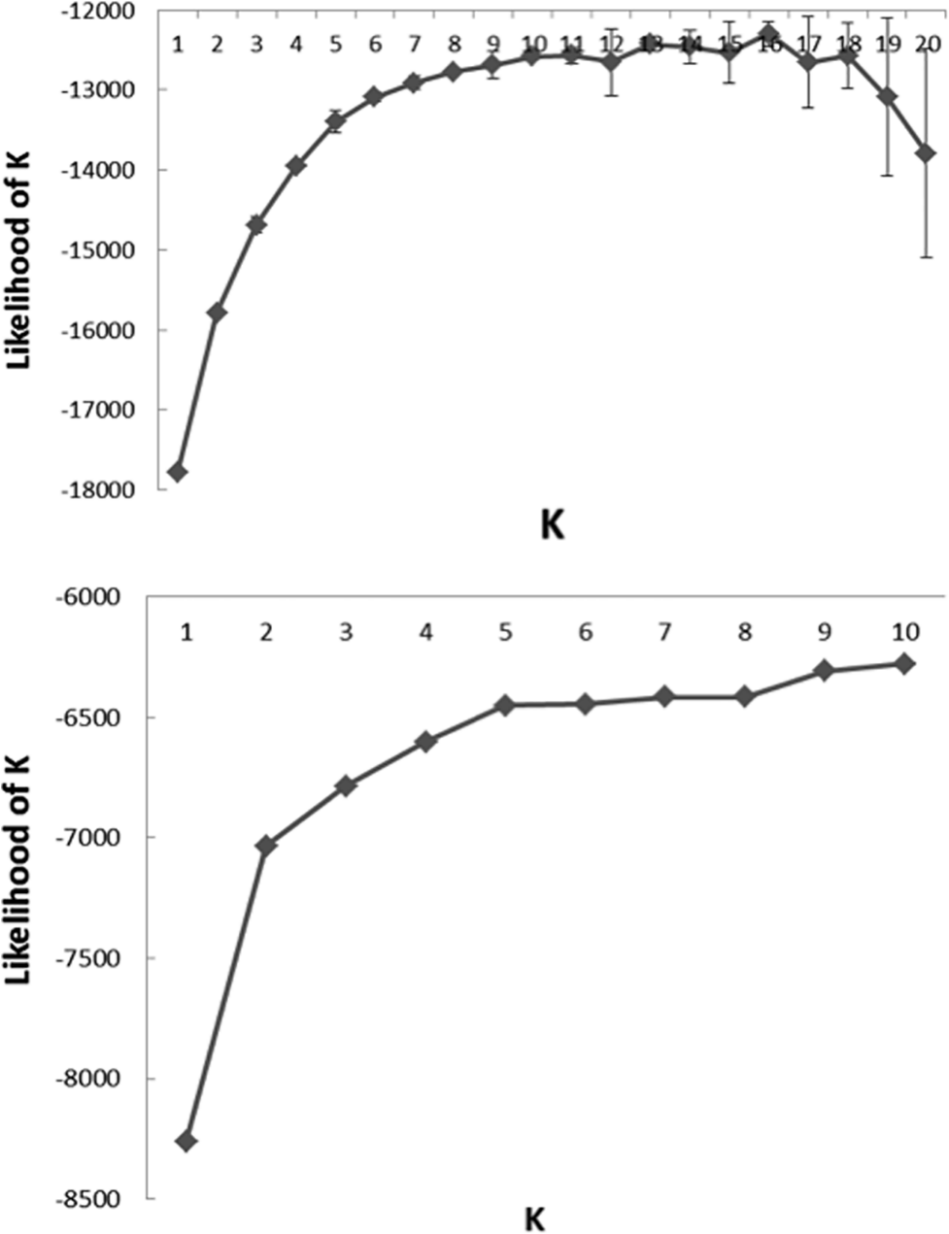
(a) The result of STRUCTURE using all samples. Average log-likelihood values over 10 runs ± SD, from 1 to 20 with all samples. (b) The result of STRUCTURE using only wild Ussrian pears in China. Average log-likelihood values over 10 runs ± SD, from 1 to 10.

**Fig 3:**
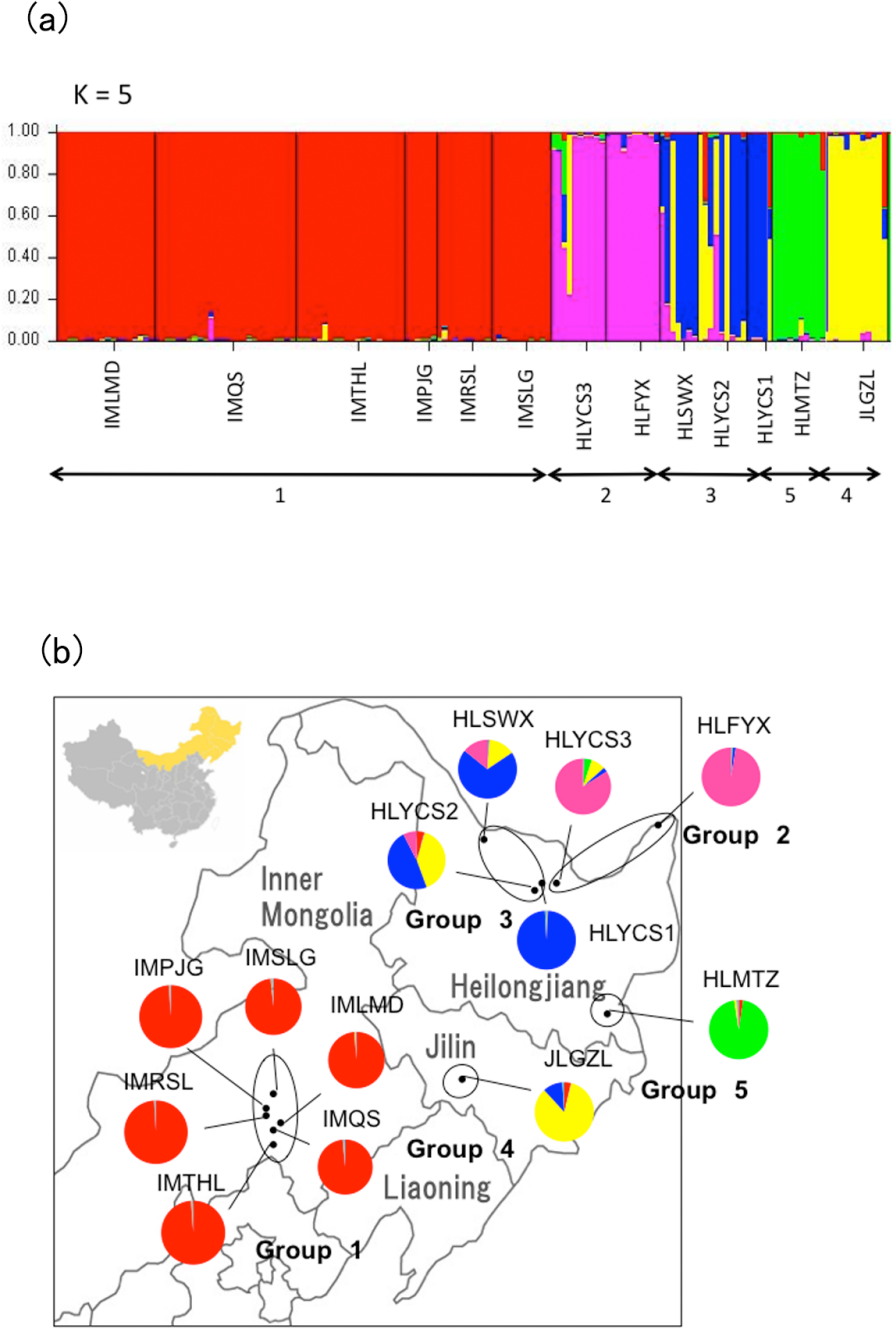
(a) Percentages of membership of genotypes to clusters (q value) inferred at K = 5 applying only wild Ussurian pears in China to STRUCTURE analysis. (b) Geographical distribution of wild populations originating from Inner Mongolia, Heilongjiang and Jilin province. Pie charts represent the proportion of gene pools based on STRUCTURE analysis when only wild Ussurian pears were applied to the analysis (K = 5).

**Fig 4:**
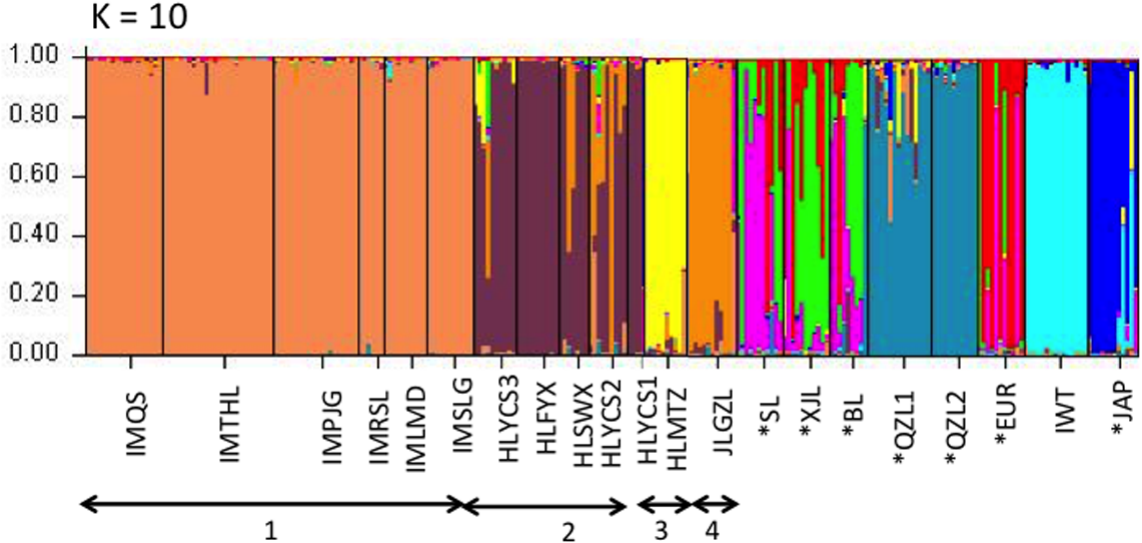
Percentages of membership of genotypes to clusters (q value) inferred at K = 10 applying all samples including wild and cultivated pears to STRUCTURE analysis. Each genotype is represented by vertical line divided into colored segments. The length of the vertical lines indicates the proportions of genome attributed to the inferred clusters. * means cultivated pears.

In the STRUCTURE analysis, the plot of the average log-likelihood values reached plateau at K = 10 (Figs 2A and 3). At K = 10, wild Ussurian pears were divided into 4 groups. But when only wild Ussurian pears were applied to STRUCTURE analysis, the average log-likelihood values reached plateau at K = 5 (Figs 2B and 4A). K = 5 reflects more reasonable result, because STRUCTURE analysis seek to identify the admixture among the species that when including the related species. So we divided wild Ussurian pears into 5 groups; (1) Inner Mongolia; IMQS, IMTHL, IMPJG, IMRSL, IMLMD, and IMSLG, (2) The north east of Heilongjiang; HLYCS3 and HLFYX, (3) the central part of Heilongjiang; HLSWX, HLYCS2 and HLYCS1, (4) Jilin; JLGZL, (5) the southern part of Heilongjiang; HLMTZ (Fig 3A). HLYCS2 was found to be admixtures of genotypes found in Jilin. Geographical distribution of 5 groups was shown in Fig 3B.

Most of the cultivars formed admixtures at K = 10, but QZL1, QZL2, and JAP were made up of relatively uniform gene pool (Fig 4).

As a result of the Mantel test, IBD pattern was detected for both nSSR and cpSSR. Pairwise *F*_*ST*_ / (1-*F*_*ST*_) values were significantly correlated with geographic distance among populations for nSSR (r = 0.687, P = 0.000) and cpSSR (r = 0.810, P = 0.000).

### Genetic diversity within populations and the Hardy-Weinberg equilibrium

For nSSRs, which harbored sufficient genetic diversity to represent the genetic variation of the *Pyrus* species (S3 Table), the average values of the indicators of na, ne, Ar, Ho, He, and *FIs* were 4.96, 2.90, 3.34, 0.40, 0.60 and 0.25 respectively for the wild Ussurian pear in China when the genetic diversity was calculated for 5 groups based on the STRUCTURE analysis (Fig 3A and 3B), and 5.85, 3.67, 1.71, 0.56, 0.71, and 0.16 respectively for Iwateyamanashi (IWT) (Table 2). These values except Ar shows that the wild Ussurian pear in China has less genetic diversity than Iwateyamanashi.

In Chinese wild populations, JLGZL showed the highest value for most of the indicators (na = 5.50, ne = 3.49, Ar = 1.61, Ho = 0.44, and He = 0.61) and HLYCS2 had the second highest values for most indicators (na = 5.00, ne = 3.46, Ar = 1.63, Ho = 0.43, and He = 0.63). All the populations from Inner Mongolia indicated low genetic diversity (na = 2.90–3.90, ne = 2.11–2.56, Ar = 1.45–1.48, Ho = 0.29–0.34, and He = 0.45–0.48) (Table 2) as did HLFYX, which come from the north east of Heilongjiang (na = 2.85, Ne = 1.99, Ar = 1.39, Ho = 0.30, and He = 0.36). The *F*_*IS*_ values were positive in all cases, except for the population, HLYCS1, and 3 populations from Inner Mongolia (IMTHL, IMPJG, and IMLMD). Two populations from Heilongjiang (HLYCS3 and HLFYX) showed significant deviations from the Hardy-Weinberg equilibrium. In cpSSRs, wild Ussurian populations from China also showed less genetic diversity than Chinese Ussurian cultivars (QZL) and Iwateyamanashi (IWT). JLGZL and HLYCS2 showed high genetic diversity, and all of the populations in Inner Mongolia had low diversity (Table 2). When the genetic diversity of wild Ussurian pear was calculated for the population based on the collection site, there was the population which had small sampling size (HLYCS1). This possibly caused minimize the values of Allelic richness, but when removed the population, HLYCS1, there was nearly unchanged in the values (S9 Table).

### Genetic relationships between wild Ussurian and cultivated pear

**Fig 5:**
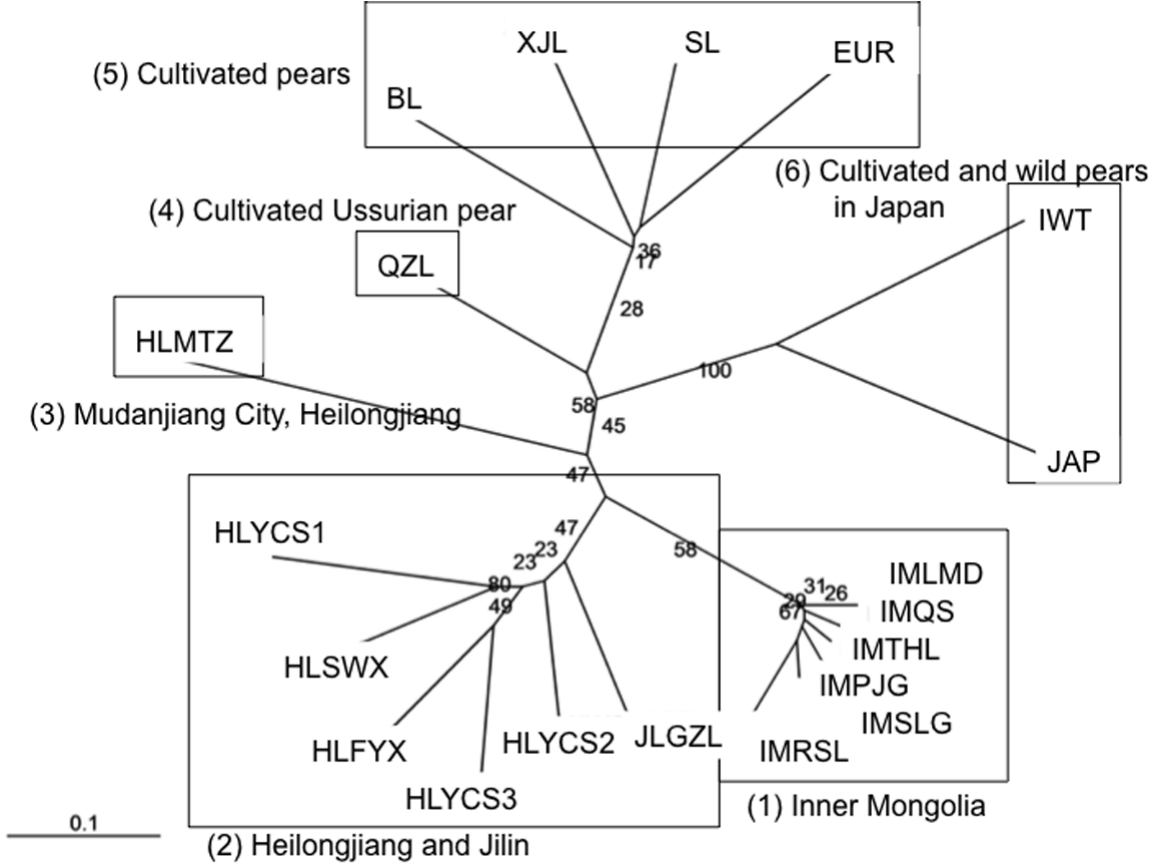
Unrooted neighbor-joining tree based on Nei’s DA distance value among populations. The number on points of divergence represents bootstrap values. (1) The 6 populations (IMQS, IMTHL, IMPJG, IMRSL, IMLMD, and IMSLG) originating from Inner Mongolia. (2) The 5 populations (HLYCS3, HLFYX, HLSWX, HLYCS2, and HLYCS1) from Heilongjiang. (3) HLMTZ from Mudanjiang, Heilongjiang. (4) The cultivated Ussurian pears. QZL1 and QZL2 were combined. (5) *P*. *pyrifolia*, *P*. *bretchneideri*, *P*. *sinkiangensis*, and *P*. *communis*. (6) Japanese cultivated pears (*P*. *pyrifolia*) and wild Ussurian pear from Japan (Iwateyamanashi).

Thirteen populations of wild Ussurian pears from China and Japan, and 6 cultivar groups were separated into 6 clades on an unrooted NJ tree; (1) The 6 populations (IMQS, IMTHL, IMPJG, IMRSL, IMLMD, and IMSLG) originating from Inner Mongolia which were all closely related. (2) The 5 populations (HLYCS3, HLFYX, HLSWX, HLYCS2, and HLYCS1) from Heilongjiang and the population JLGZL from Jilin also showed close relationships. Group (3) including HLMTZ from Mudanjiang, Heilongjiang was distantly related the other populations from Heilongjiang, and was positioned near the cultivated Ussurian pear group (4). Group (5) contained the other cultivated pears such as *P*. *pyrifolia*, *P*. *bretchneideri*, *P*. *sinkiangensis*, and *P*. *communis*. Japanese cultivated pears (*P*. *pyrifolia*) and wild Ussurian pear from Japan (Iwateyamanashi) were positioned far away from the other groups (6) (Fig 5).

### Flower and fruit morphology

**Fig 6:**
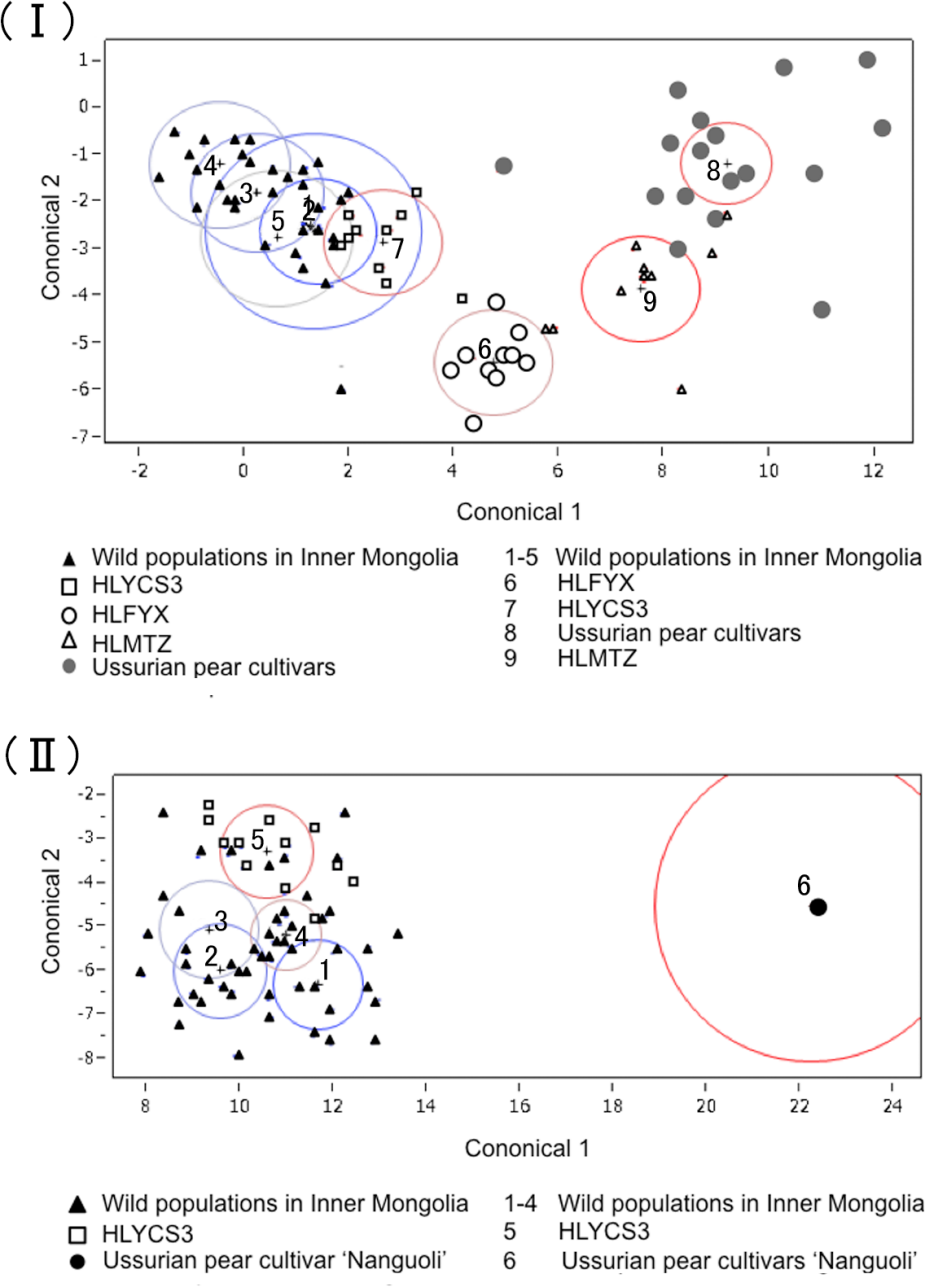
Discriminant analysis for canonical 1 and 2. (Ⅰ) represented flower morphologies measured from 9 wild populations; Inner Mongolia (1:IMQS, 2: IMTHL, 3:IMPJG, 4:IMLMD, and 5:IMSLG), Heilongjiang (7:HLYCS, 6:HLFYX, and 9:HLMTZ), and 26 Ussurian pear cultivars (8). (Ⅱ) represented fruit morphologies, containing 5 wild populations; Inner Mongolia (1:IMQS, 2:IMTHL, 3:IMPJG, and 4:IMSLG), Heilongjiang (5:HLYCS), and an Ussurian pear cultivar ‘Nanguoli’(6).

In order to demonstrate the relationships between populations based on flower and fruit morphologies, discriminant analysis was carried out using the values of 5 floral and 4 fruit morphological characters (Fig 6I and 6II). The 5 populations from Inner Mongolia (IMQS, IMTHL, IMPJG, IMLMD, and IMSLG) was located on the left side and closely related each other. The individuals from HLYCS were plotted near the Inner Mongolian populations. HLFYX, HLMTZ and Ussurian pear cultivars were located separately (Fig 6I). The individuals plotted on left upper side such as 5 populations in Inner Mongolia and HLYCS tended to have small flowers, short peduncles, and slender petals. In contrast, most of the cultivars, had large flowers, long peduncles, and round petals. The flower shape in the population ‘HLFYX’ and ‘HLMTZ’ was of intermediate flower morphology harboring relatively big flowers and long peduncles. For fruit morphology, IMQS, IMTHL, IMPJG, and IMSLG positioned left lower part (Fig 6II). HLYCS3 was plotted on left upper side. An Ussurian pear cultivar ‘Nanguoli’ was located on the right middle side. The fruit size and peduncle length of ‘Nanguoli’ were bigger and longer than those of wild Ussurian pears. With regard to wild populations, all of the fruits were much smaller than that of ‘Nanguoli’. The fruit morphologies of wild Ussurian pears could be divided into two groups: circle (1) the group plotted on left lower side such as Mongolian populations harboring globular shape and short peduncles, and circle (2) the group located on left upper part such as HLYCS3 harboring ellipse shape and relatively long peduncles.

## Discussion

### Low genetic diversity of wild Ussurian pears

Using nSSR markers, we have shown that wild Ussurian pears from China have much lower genetic diversity Iwateyamanashi. The genetic diversity of wild Ussurian pear in China inferred from the values of na, ne, Ho, and He was low compared to the values of wild grapevine from Spain, wild apple (*Malus sieversii* and *M*. *sylvestris*), African fruit tree of Rosaceae (*Prunus africana*), wild Callery pear (*P*. *calleryana*), and the average of many perennial and outcross species [52, 53, 54, 55, 56].

For cpSSRs, the genetic diversity within each wild Ussurian pear population was lower than that of the other trees such as *Ainsliaea faurieana* (*Asteraceae*) on Yakushima Island, Japan, *Quercus semiserrata* Roxb. in northern Thailand, *Pinus albicaulis* Engelm. (whitebark pine), Mexican pinyon pine (*Pinus nelsonii* Shaw), *Magnolia stella*, and Apricot [57, 58, 59, 60].

The low genetic diversity of Chinese wild Ussurian pears might be the result of restrictive gene flow which is important in maintaining genetic diversity and minimizing genetic drift arising from habitat fragmentation because of deforestation caused by climate change in Inner Mongolia and human development in Heilongjiang. Pairwise *F*_*ST*_ were also significantly different among populations with the exception of several populations within Inner Mongolia (S3 Table and S4 Table).

The measurement of within population diversity revealed that the values of *F*_*IS*_ were high in most of the wild populations in China (Table 2). This result could be attributed to nonrandom mating promoted by habitat fragmentation and limited gene flow causing inbreeding. S-locus usually can avoid self-pollination and crossing closely-related individuals, but when belonging to small population, inbreeding such as sister brother mating can be occurred.

### Genetic population structure in wild Ussurian pears in China

Wild Ussurian pears in China were highly structured. Differentiation among collecting sites was higher than other congeneric species (20.02%), referring to previous studies such as *P*. *calleryana*: 8.86% for among populations, 91.14% for within a population [61], *P*. *ussuriensis* var. *aromatica*: 7.20% for among populations, 92.80% for within a population (S6 Table). For cpSSRs, genetic variations among populations of wild Ussurian pears (44.40%) from China were twice higher than that of wild Callery pears (22.9%) from China [61]. These results indicated that the wild Ussurian pears in China had high population structure. Actually, wild Ussurian pear in China divided into 5 groups based on the STRUCTURE analysis and chloroplast haplotype distribution (Figs 1A and 4B). The grouping using nSSRs and cpSSRs almost corresponded except north of Heilongjiang. STRUCTURE analysis divided the populations in north of Heilongjiang into 2 groups (Group2; HLYCS3 and HLFYX, Group3; HLYCS1, HLYCS2, and HLSWX). The populations in north of Heilongjiang such as HLYCS1, HLYCS2, and HLSWX were belonging to the same group by STRUCTURE analysis, but in chloroplast haplotype distribution HLYCS1 was unique and didn’t have haplotype E which was representative haplotype in Heilongjiang. HLSWX, HLFYX, and HLYCS3 belonging to green colored Group 2 by cpSSRs locate near the river, the first 2 populations are in close to Amur River and the latter is in close vicinity to Tangwang River flowing into Songhua River which is the greatest tributary of Amur River (Fig 1A). The circumstances of these 3 populations are similar, such as woody and damp ground. However, in STRUCTURE analysis (K = 5) most of the individuals from HLSWX were assigned to dark blue colored cluster same as HLYCS1 and HLYCS2 (Fig 3A and 3B). HLSWX, HLYCS1, and HLYCS2 are located on skirt of Xiaoxinganling Mountains, and therefore gene flow by pollen may often occur among these 3 populations. HLYCS3 also close to location of HLYCS1 and HLYCS2, however, HLYCS3 is located on small woods separated from Xiaoxinganling Mountains. Thus the gene flow might not be so frequent between HLYCS3 and HLYCS1 and HLYCS2. By taking into account geographic factor such as mountains, 5 groups revealed by STRUCTURE analysis are suitable for representing current population structure of wild Ussurian pear in China (Fig 3B).

A significant correlation between genetic distance and geographic distance was detected by a Mantel test using both nSSRs and cpSSRs. Isolation by distance was expected to be the main factor causing the high level of population divergence in wild Ussurian pears, due to limited gene flow and the effects of genetic drift as a result of habitat fragmentation. Geographic distance influences migration of pollen and seed and restricts the gene flow among populations. The ratio of pollen to seed flow was calculated using the equation of Ennos [45], and the result showed that pollen flow was 5.8 times higher than seed flow. The ratios reported previously for other tree species showed much higher values than that for wild *P*. *ussuriensis* from China (*Quercus petraea*; 196: 1, *Pinus contorta*; 28: 1, *P*. *radiate*; 31: 1, *P*. *attenuat*; 44: 1, *P*. *muricata*; 24: 1) [45]. This is mainly caused by the activities of frugivorous animals. Small pear fruits are favored by many kinds of animals, and the seeds are carried relatively long distances. A similar result was obtained for wild *P*. *calleryana* from China [61]. The ratio of pollen to seed was 0.56: 1, this means that seed flow plays an important role in wild callery pear trees. It is thought that wild Ussurian pear seed could also be moved easily.

‘Group 1’ included all of the populations from Inner Mongolia (Fig 3B). Wild Inner Mongolian populations were genetically and morphologically distant from other wild populations (Figs 1A, 4A and 5). The analysis of within population diversity revealed that the 6 populations from Inner Mongolia, in particular, showed lower genetic diversity than the other populations of Chinese Ussurian pears. This peculiarity of the Inner Mongolian populations is explained by the following: (1) Isolation by distance (IBD); the Mantel test detected significant IBD patterns for both nSSRs and cpSSRs. The six populations from Inner Mongolia are located far from the other wild populations. Therefore the gene flow between the Inner Mongolian populations and the other wild populations is reduced because of their geographic separation, and so the wild populations in Inner Mongolia have evolved different genotypes to the other wild populations. (2) Bottleneck effect: the wild populations in Inner Mongolia have been conjectured that they were experienced bottleneck from the demographic reduction by environmental changes, so that inbreeding has occurred in small populations leading to uniform genotype and low genetic diversity.

‘Group 5’ included HLMTZ, from Muling, Mudanjiang City, Heilongjiang Province. Based on STRUCTURE analysis, this population was separated from the other wild populations in Heilongjiang, and positioned near the Ussurian pear cultivar group in the phylogenic tree (Fig 5). Moreover the flower morphologies of this population were similar to those of the Ussurian pear cultivars (Fig 6). Historically, cultivation of Ussurian pear was prosperous in Mudanjiang City, so introgression of Ussurian pear cultivars into wild Ussurian pear trees might have occurred frequently in HLMTZ over time. This was also known from low bootstrap value in the phylogenetic tree (Fig 5). This result is consistent with the previous reports that *P*. *ussuriensis* growing wild in Northern part of Japan was introgressed from Japanese pear cultivars [17, 62].

### The inference of primitive haplotype of wild Ussurian pear in China

Haplotype E which is the representative haplotype of Heilongjiang, and according to coalescent theory [63], there is a possibility that haplotype E is primitive haplotype detected in this study, because it shows the largest number of connections with other haplotypes including wild and cultivated Ussurian pears (S1 Fig) and positioned in the center of the network. Previous research by Wuyun *et al*. [11] using hypervariable regions of cpDNA also revealed that a haplotype (Hcp3) found in populations such as HLSWX, HLYCS3, HLFYX, and JLGZL in Heilongjiang and Jilin was at the divergent center of a haplotype network. Therefore, haplotype E is very important to investigate the origin of Ussurian pear.

### The inference of domestication of Ussurian pear

Although the flower shape in the population ‘HLFYX’ represented intermediate flower morphology between wild and its derived cultivars, wild Ussurian pear and cultivated Ussurian pear in China were genetically divergent from each other (Figs 4, 5 and 6I). Another study by Cao *et al* [21] using M13-tailed SSR markers showed that Ussurian pear cultivars had close relationships with the wild accessions found in the southern areas of north east of China, such as Liaoning and Hebei. All of the wild Ussurian pear trees from Heilongjiang and Jilin were clustered in a group by UPGMA tree, and were separate from the cluster of Ussurian pear cultivars [21]. From molecular data, wild pears in Heilongjiang and Jilin were not considered to be related with domestication of cultivated Ussurian pear. More cultivated Ussurian pears need to be analysed to know the origin of Ussurian cultivars.

The phylogenetic relationship of Ussurian pear in China and Iwateyamanashi

In this study, *P*. *ussuriensis* Maxim. (wild Ussurian pear in China) and *P*. *ussuriensis* var. *aromatica* (Iwateyamanashi) were genetically divergent from the result of STRUCTURE analysis and phylogenetic tree (Figs 4 and 5). This reason is as follows; Most of the oldest definitive members of the Rosaceae were already present in the Eocene upland floras of the Okanogan Highlands of northeastern Washington State and British Columbia, Canada [64]. The fossils of leaves recognized as *Pyrus* were also discovered in the stratum of Eocene [65]. Neogene rosaceous occurrences are more widespread with reports known in Asia, particularly Japan, the Arctic, several Gondwanan regions, and northern Africa [64]. At that time the Japanese archipelago have been connected with the Asian continent by land deformation, and *P*. *ussuriensis* Maxim. was considered to be already distributed in the region which was the present northern part of Japan and northeast part of Eurasian continent. From the latter Miocene to the Pliocene, the Sea of Japan became to expand and the original form of present island of Japan were developed, consequently both of *P*. *ussuriensis* Maxim. in Japan and continent were geographically isolated. There was little gene flow between *P*. *ussuriensis* Maxim. (wild Ussurian pear in China) and *P*. *ussuriensis* var. *aromatica* (Iwateyamanashi), then each of these *Pyrus* followed the evolutionally different way.

### Conservation strategy

The detection of population differentiation using cpSSRs haplotypes and STRUCTURE analysis using nSSRs can help to define appropriate conservation units, and provide a good focus for conservation management [66]. Wild Ussurian pear in China can be divided into 5 genetically distinct groups (Fig 3A and 3B), and their flower and fruit morphologies diverged in each population (Fig 6I and 6II). But group 5 was considered to be the population introgressed by cultivars, so 4 conservation units were set up with removing group 5.

Group 2 and 3 had high genetic diversity. In Group 2, the genetic diversity of HLYCS2 having rich ecological conditions which included all of the genotypes in Heilongjiang revealed by STRUCTURE analysis was especially high. Conservation priority should be given to populations with a high level of genetic diversity and unique genotypes possessing the risk of extinction in the future. HLYCS2 should be perfectly conserved for *in situ* level. This study also revealed that the genetic diversity of group 1 was especially low (Table 2). In these populations, inbreeding and the shortage of gene flow were indicated (Table 2, Figs 3A and 5), thus the risk of extinction in the future was high. Because some individuals in Inner Mongolian populations had unique genotypes, these individuals should be reserved with *ex situ* conservation.

## Supporting Information

S1 Fig Median-joining network for 51 chloroplast SSR haplotypes. These haplotypes were detected from 124 individuals in Chinese wild Ussurian pears (A-Y), 10 individuals of Iwateyamanashi (Z, a-i), and 16 individuals of cultivated Ussurian pears in China (AA-PP). The haplotypes are indicated by yellow circles, and small red circles show median vectors. The size of each pie chart is proportional to the frequency of corresponding haplotype.
(TIF)

S1 Table Characteristics of 20 nSSR and 16 cpSSR markers used in this study. (DOCX)

S2 Table Summary statistics for the 20 nSSR markers in *P*. *ussuriensis* Maxim. (DOCX)

S3 Table Summary statistics for the 16 cpSSR markers in *P*. *ussuriensis* Maxim. (DOCX)

S4 Table Pairwise genetic differentiation (*F*_*ST*_) among the prior populations using nSSRs. (DOCX)

S5 Table Pairwise genetic differentiation (*F*_*ST*_) among the prior populations using cpSSRs. (DOCX)

S6 Table Analysis of molecular variance (AMOVA) averaged across 20 nSSR using 275 individuals of Iwateyamanashi (*P*. *ussuriensis* Maxim. var *aromatica*) from north east Japan. (DOCX)

S7 Table The characteristic of floral morphologies for wild *P*. *ussuriensis* Maxim in Inner Mongolia, Heilongjiang, and Ussurian pear cultivars. (DOCX)

S8 Table The characteristics of fruit morphologies for wild *P*. *ussuriensis* Maxim. in Inner Mongolia, Heilongjiang, and an Ussurian pear cultivar ‘Nanguoli’. (DOCX)

S9 Table Allelic richness with and without HLYCS1 in *P*. *ussuriensis*. (DOCX)
