# Supplementary figures and images for: Population Structure of and Conservation Strategies for Wild Pyrus ussuriensis Maxim. in China
Source: PLoS One. 2015 Aug 7;10(8):e0133686. doi: 10.1371/journal.pone.0133686 (PMC4529180; doi:10.1371/journal.pone.0133686)

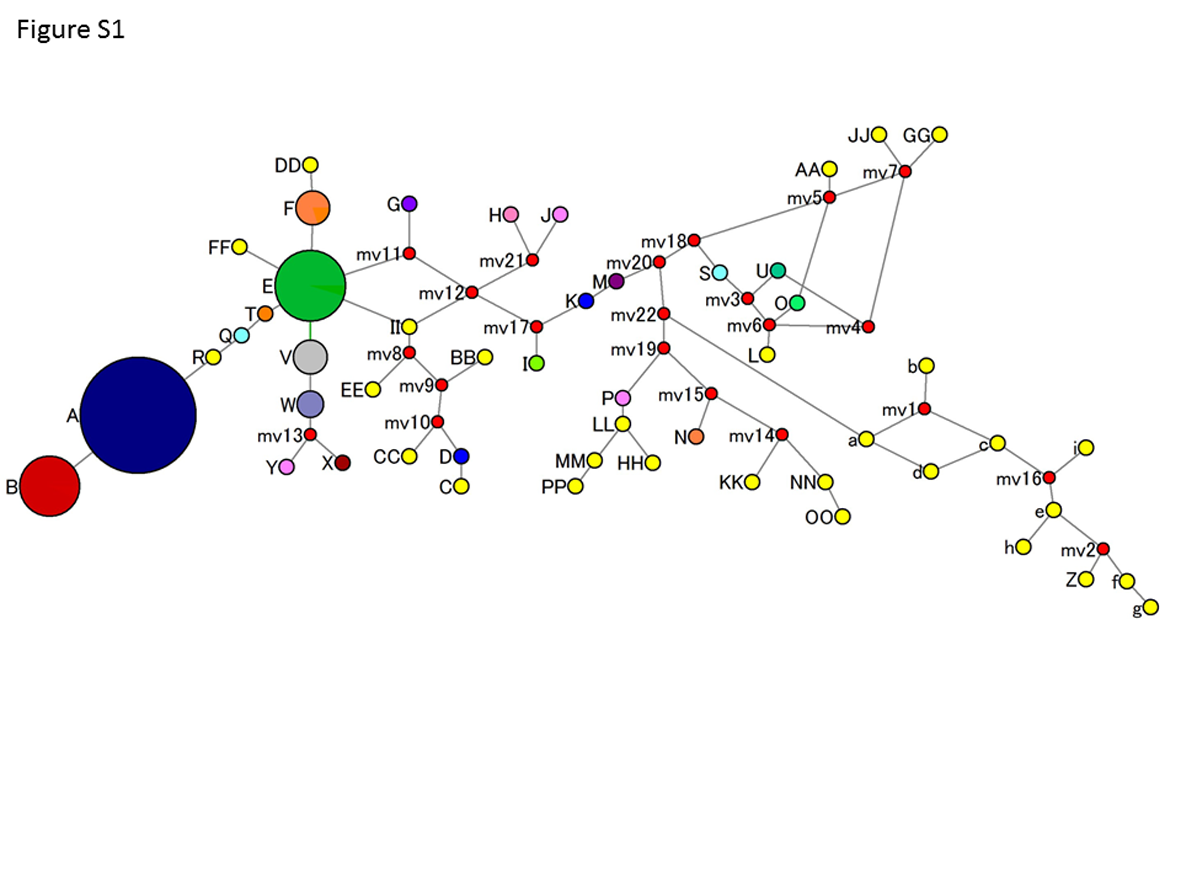

Supplement: S1 Fig — These haplotypes were detected from 124 individuals in Chinese wild Ussurian pears (A-Y), 10 individuals of Iwateyamanashi (Z, a-i), and 16 individuals of cultivated Ussurian pears in China (AA-PP). The haplotypes are indicated by yellow circles, and small red circles show median vectors. The size of each pie chart is proportional to the frequency of corresponding haplotype. (TIF) [file pone.0133686.s001.tif]
