# Supplementary material for: Population Structure of and Conservation Strategies for Wild Pyrus ussuriensis Maxim. in China
Source: PLoS One. 2015 Aug 7;10(8):e0133686. doi: 10.1371/journal.pone.0133686 (PMC4529180; doi:10.1371/journal.pone.0133686)
Supplement: S1 Table — (DOCX) [file pone.0133686.s002.docx]

S1 Table. Characteristics of 20 nSSR and 16 cpSSR markers used in this study

| SSR locus | Origin | Primer sequence (5'-3') | Motif | Linkage group*(Position) | Reference |
| --- | --- | --- | --- | --- | --- |
| Nuclear |  |  |  |  |  |
| TsuENH155 | Hosui | F : CGACTCTCCCTCACTTTTGC |  |  | Nishitani *et al*. 2005 |
|  |  | R : GTTTCTTGGAGAAATGGATCTCAAGCG |  |  |  |
| NH029a | Hosui | F : GAAGAAAACCAGAGCAGGGCA | (AG)8 | Ba9, La9 | Yamamoto *et al.* 2002a |
|  |  | R : CCTCCCGTCTCCCACCATATTAG |  |  |  |
| BGA35 | Bartlet | F : AGAGGGAGAAAGGCGATT | (AG)8 | La3 | Yamamoto *et al.* 2001 |
|  |  | R : GTTTCTTGCTTCATCACCGTCTGCT |  |  |  |
| NB104a | Bartlet | F : TCGGAGAGGAAGAGTTGGAGGA | (GT)3GC(GT)5(GA)2TA(GA)5 | La12 | Sawamura *et al.* 2004 |
|  |  | R : AGGTCCGTGCCCAGTTTCTTTC |  |  |  |
| CH02e02 | Apple | F : CTCATCAGTCTCACTGACTGTGTG |  | Ho13, La13 | Liebhard *et al.* 2002 |
|  |  | R : GTTTCTTAGGGTCAGGGTCAGTCAGG |  |  |  |
| CH02b10 | Apple | F : CAAGGAAATCATCAAAGATTCAAG | (GA)19.5 | Ba2, Ho2, La2 | Gianfranceschi *et al.* 1998 |
|  |  | R : CAAGTGGCTTCGGATAGTTG |  |  |  |
| NB105a | Bartlet | F : AAACAACCGACTGAGCAACATC | (AG)15AT(AG)10 | Ba11, La11 | Yamamoto *et al*. 2002b |
|  |  | R : AAAATCTTAGCCCAAAATCTCC |  |  |  |
| NH009b | Hosui | F : CCGAGCACTACCATTGA | (AG)20 | Ba13, La13 | Yamamoto *et al*. 2002a |
|  |  | R : GTTTCTTCGTCTGTTTACCGCTTCT |  |  |  |
| NB141b | Bartlet | F : GTTTCTTCAGAGAAAGACAGAGGTAGAGAGAA | (AT)8(AG)15.5 | Ba4, La4 | Sawamura *et al.* 2004 |
|  |  | R : GGATTGATCGCCTTATGGTTGT |  |  |  |
| CH02d10b | Apple | F : GTAACCTTTGTTGCGCGTGG |  | Ba15, Ho15, La15 | Liebhard *et al.* 2002 |
|  |  | R : GTTTCTTGCCTTGAGTTTCTCAGCATTG |  |  |  |
| CH03g06 | Apple | F : ATCCCACAGCTTCTGTTTTTG |  | Ba14, Ho14, La14 | Liebhard *et al.* 2002 |
|  |  | R : TCACAGAGAATCACAAGGTGGA |  |  |  |
| NH039a | Hosui | F : TGGTTGCCGAGAAAGTGTAG | (GA)6CA(GA)9.5 | Ba10, Ho10, La10 | Sawamura *et al.* 2004 |
|  |  | R : CAAGCAAGTACAACATGAGTGG |  |  |  |
| CH02b03b | Apple | F : ATAAGGATACAAAAACCCTACACAGG |  | Ba10, Ho10, La10 | Liebhard *et al.* 2002 |
|  |  | R : GACATGTTTGGTGGTTGAAAACTTG |  |  |  |
| NH206a | Hosui | F : AGAGATGGAACTTTATATGTATGTGTG | (AC)_9.5_ | Ho10, La10 | Sawamura *et al.* 2004 |
|  |  | R : GTTTCTTCCCCTTGTTTTTTTTATGTTTG |  |  |  |
| NB109a | Bartlet | F : ATGCTCTATAAAACCCACCTACC | (AG)_18_ | Ba3, Ho3, La3 | Yamamoto *et al*. 2002b |
|  |  | R : AGAGGGACCATTGTGTTATTGTAT |  |  |  |
| NH203a | Hosui | F : TCGATACTCCACAAGACTGCTC | (AC)_7.5_ | Ba3, Ho3, La3 | Sawamura *et al.* 2004 |
|  |  | R : GTTTCTTCCACCTCCAAGCTCAAGTTTC |  |  |  |
| EMPc114 | Doyenne du Comice | F : GTACCCACAATTCCCCATAT | (AG)20 | Ba10, Ho10 | Fernandez *et al*. 2006 |
|  |  | R : GTTTCTTAGCCTTATGCGCCTTCTACC |  |  |  |
| CH04g12 | Apple | F : CACCGATGGTGTCAACTTGT |  | Ba8, Ho8, La8 | Liebhard *et al.* 2002 |
|  |  | R : GTTTCTTCAACAAAATGTGATCGCCAC |  |  |  |
| CH02g01 | Apple | F : GATGACGTCGGCAGGTAAAG |  | Ho13, La13 | Liebhard *et al.* 2002 |
|  |  | R : GTTTCTTCAACCAACAGCTCTGCAATC |  |  |  |
| CH03d10 | Apple | F : CTCCCTTACCAAAAACACCAAA |  | Ba2, Ho2, La2 | Liebhard *et al.* 2002 |
|  |  | R : GTTTCTTGTGATTAAGAGAGTGATCGGGG |  |  |  |
| Chloroplast |  |  |  |  |  |
| Pchssr-3 | Hosui | F : ACCCATGTCAACCAATACCG | (T)11 | 1599 (psbA-tmk-UUU) | in this study |
|  |  | R : ATCCGATAGTTCCGGGTTC |  |  |  |
| Pchssr-6 | Hosui | F : TCGGGTCATAAAAACCCACT | (C)10 | 54441 (trnK UUU-rsp16) | in this study |
|  |  | R : GCCGTACGAGGAGAAAACTTC |  |  |  |
| Pchssr-14 | Hosui | F : AATGCCATCGCCTACTTGAA | (T)11 | 12515 (atpF) | in this study |
|  |  | R : GGTATGTTTGGAACGGTGAAA |  |  |  |
| Pchssr-17 | Hosui | F : CCCACAAGGATAGGTACAATCAA | (A)12 | 15309 (atpH-atpI) | in this study |
|  |  | R :ACGACGTGCGATTTTTATGG |  |  |  |
| Pchssr-19 | Hosui | F : TGCTTCCATCATCTCTTCCA | (T)11 | 17410 (atpI-rps2) | in this study |
|  |  | R : TGTATCAACGGCCAATTTCC |  |  |  |
| Pchssr-27 | Hosui | F : CAAGCATGCCCCTTTGTAA | (T)10 | 38621 (psbZ-tmG GCC) | in this study |
|  |  | R : AAACAGCCAATTGGAAAGCA |  |  |  |
| Pchssr-31 | Hosui | F : GGCCTGCCCTAAGAATAACA | (A)19 | 50289 (trnL UAA) | in this study |
|  |  | R : TGCCCCTATTTCTACCGTTT |  |  |  |
| Pchssr-36 | Hosui | F : GCTGGTTGATGAGAGTTACTTCG | (A)11 | 63164 (psaI-rcf4) | in this study |
|  |  | R : TTTCGAGACCCCGCTATAAG |  |  |  |
| Pchssr-39 | Hosui | F : CTCATATTCTTCTTACGATTCAATTAGACG | (T)13 | 68391 (psbE-petL) | in this study |
|  |  | R : GGTGGCCAACCAATTCCT |  |  |  |
| Pchssr-42 | Hosui | F : ATCCCTACCCCTCCCTTETT | (A)15 | 70641 (psaJ-rp133) | in this study |
|  |  | R : GATATTGTTGCCCCAACCAC |  |  |  |
| Pchssr-44 | Hosui | F : TATAACCTTCCCGACCACGA | (T)18 | 72500 (rpl20-rps12 5end) | in this study |
|  |  | R : AGGGCTCCGGTGTATAGAGA |  |  |  |
| Pchssr-45 | Hosui | F : ATGATGGCTCCGTTGCTTTA | (T)10 | 73965 (clpP1) | in this study |
|  |  | R : GAGGCCTATTTCAGCGTCAC |  |  |  |
| Pchssr-48 | Hosui | F :GGGGCTTTTCTTTCATTTTTC | (A)11 | 74801 (clpP1) | in this study |
|  |  | R : GAAGCCCATTCAGGAACAAGAG |  |  |  |
| Pchssr-50 | Hosui | F: ACTCCCTTGGGGATTCTTCTACG | (T)19 | 83026 (rps11-rpl36) | in this study |
|  |  | R : CGAAATCTAAAGGACCCGATG |  |  |  |
| Pchssr-55 | Hosui | F : TTGTTTCTGAGTCACCGGTTC | (A)10 | 116531 (ndhF-rpl32) | in this study |
|  |  | R : TCATTTGACCAATTATAACCTCTTG |  |  |  |
| Pchssr-60 | Hosui | F : AATAACCCGTTGGTTTCACG | (T)11 | 125692 (ndhA) | in this study |
|  |  | R : TTGAAAGCCCTCTTTTTCTCAG |  |  |  |
