# Supplementary material for: Population Structure of and Conservation Strategies for Wild Pyrus ussuriensis Maxim. in China
Source: PLoS One. 2015 Aug 7;10(8):e0133686. doi: 10.1371/journal.pone.0133686 (PMC4529180; doi:10.1371/journal.pone.0133686)
Supplement: S2 Table — (DOCX) [file pone.0133686.s003.docx]

S2 Table. Summary statistics for the 16 cpSSR markers in *P. ussuriensis* Maxim.

| Locus | Number of allels | ne | h |
| --- | --- | --- | --- |
| Pchssr-3 | 9 | 3.107 | 0.678 |
| Pchssr-19 | 7 | 2.971 | 0.663 |
| Pchssr-36 | 4 | 2.304 | 0.566 |
| Pchssr-60 | 6 | 2.053 | 0.513 |
| Pchssr-27 | 7 | 3.413 | 0.707 |
| Pchssr-45 | 7 | 3.581 | 0.721 |
| Pchssr-14 | 4 | 2.480 | 0.597 |
| Pchssr-44 | 10 | 3.523 | 0.716 |
| Pchssr-50 | 9 | 4.138 | 0.758 |
| Pchssr-17 | 3 | 2.206 | 0.547 |
| Pchssr-27 | 2 | 1.048 | 0.046 |
| Pchssr-48 | 3 | 2.189 | 0.543 |
| Pchssr-42 | 3 | 1.704 | 0.413 |
| Pchssr-31 | 8 | 3.245 | 0.692 |
| Pchssr-6 | 4 | 1.262 | 0.207 |
| Phssr-39 | 5 | 1.496 | 0.332 |
| Average | 5.7 | 2.545 | 0.544 |

ne: effective number of alleles (Kimura & Crow 1964), h:haplotype diversity.
