# Supplementary material for: Population Structure of and Conservation Strategies for Wild Pyrus ussuriensis Maxim. in China
Source: PLoS One. 2015 Aug 7;10(8):e0133686. doi: 10.1371/journal.pone.0133686 (PMC4529180; doi:10.1371/journal.pone.0133686)
Supplement: S3 Table — (DOCX) [file pone.0133686.s004.docx]

S3 Table. Summary statistics for the 20 nSSR markers in *P. ussuriensis* Maxim.

| Name | Number of allele | ne | He | Ho | F_IS_ |
| --- | --- | --- | --- | --- | --- |
| TsuENH155 | 15 | 0.785 | 0.744 | 0.161 | 0.653 |
| NH029a | 14 | 6.785 | 0.854 | 0.747 | -0.036 |
| BGA35 | 11 | 3.469 | 0.713 | 0.207 | 0.572 |
| NB104a | 12 | 1.581 | 0.368 | 0.198 | 0.255 |
| CH02e02 | 8 | 1.139 | 0.122 | 0.103 | 0.038 |
| CH02b10 | 16 | 1.827 | 0.454 | 0.255 | 0.172 |
| NB105a | 16 | 7.051 | 0.860 | 0.479 | 0.297 |
| NH009 | 15 | 6.161 | 0.839 | 0.389 | 0.460 |
| NB141 | 26 | 7.361 | 0.866 | 0.566 | 0.140 |
| CH02d10b | 27 | 2.508 | 0.602 | 0.317 | 0.189 |
| CH03g06 | 27 | 4.633 | 0.786 | 0.602 | -0.077 |
| NH039a | 22 | 5.380 | 0.816 | 0.574 | 0.126 |
| CH02b03 | 18 | 2.234 | 0.553 | 0.213 | 0.308 |
| NH206a | 27 | 8.143 | 0.879 | 0.295 | 0.547 |
| NB109a | 39 | 9.334 | 0.895 | 0.458 | 0.415 |
| NH203a | 20 | 6.224 | 0.841 | 0.599 | 0.097 |
| EMPc114 | 14 | 2.671 | 0.627 | 0.233 | 0.636 |
| CH04g12 | 27 | 13.167 | 0.926 | 0.608 | 0.215 |
| CH02g01 | 17 | 5.758 | 0.828 | 0.508 | 0.214 |
| CH03d10 | 23 | 7.870 | 0.875 | 0.609 | 0.157 |
| Average | 19.7 | 5.204 | 0.722 | 0.406 | 0.269 |

ne: effective number of alleles (Kimura & Crow 1964),

Ho: observed heterozygosity, He: expected heterozygosity,

F_IS_: fixtation index
