# Supplementary material for: Population Structure of and Conservation Strategies for Wild Pyrus ussuriensis Maxim. in China
Source: PLoS One. 2015 Aug 7;10(8):e0133686. doi: 10.1371/journal.pone.0133686 (PMC4529180; doi:10.1371/journal.pone.0133686)
Supplement: S4 Table — (DOCX) [file pone.0133686.s005.docx]

S4 Table. Pairwise genetic differentiation (*F_ST_*) among the prior populations

|  | IMQS | IMTHL | IMPJG | IMRSL | IMLMD | IMSLG | HLYCS1 | HLYCS2 | HLYCS3 | HLFYX | HLSWX | JLGZL | HLMTZ |
| --- | --- | --- | --- | --- | --- | --- | --- | --- | --- | --- | --- | --- | --- |
| IMQS | 0.000 | * | * | - | - | ** | ** | ** | ** | ** | ** | ** | ** |
| IMTHL | 0.024 | 0.000 | ** | ** | * | ** | ** | ** | ** | ** | ** | ** | ** |
| IMPJG | 0.030 | 0.042 | 0.000 | - | ** | ** | ** | ** | ** | ** | ** | ** | ** |
| IMRSL | 0.034 | 0.077 | 0.044 | 0.000 | - | ** | ** | ** | ** | ** | ** | ** | ** |
| IMLMD | 0.000 | 0.042 | 0.049 | 0.058 | 0.000 | ** | ** | ** | ** | ** | ** | ** | ** |
| IMSLG | 0.060 | 0.613 | 0.062 | 0.082 | 0.052 | 0.000 | ** | ** | ** | ** | ** | ** | ** |
| HLYCS1 | 0.218 | 0.232 | 0.235 | 0.245 | 0.206 | 0.305 | 0.000 | ** | ** | * | ** | ** | ** |
| HLYCS2 | 0.132 | 0.160 | 0.155 | 0.123 | 0.123 | 0.196 | 0.041 | 0.000 | ** | ** | ** | ** | ** |
| HLYCS3 | 0.237 | 0.237 | 0.233 | 0.226 | 0.224 | 0.281 | 0.144 | 0.078 | 0.000 | ** | ** | ** | ** |
| HLFYX | 0.268 | 0.257 | 0.250 | 0.267 | 0.248 | 0.311 | 0.178 | 0.126 | 0.094 | 0.000 | ** | ** | ** |
| HLSWX | 0.178 | 0.194 | 0.192 | 0.188 | 0.170 | 0.235 | 0.102 | -0.002 | 0.124 | 0.169 | 0.000 | ** | ** |
| JLGZL | 0.140 | 0.164 | 0.168 | 0.135 | 0.141 | 0.204 | 0.072 | 0.033 | 0.192 | 0.172 | 0.083 | 0.000 | ** |
| HLMTZ | 0.280 | 0.257 | 0.257 | 0.274 | 0.306 | 0.293 | 0.380 | 0.251 | 0.261 | 0.339 | 0.310 | 0.107 | 0.000 |
