# Supplementary material for: Population Structure of and Conservation Strategies for Wild Pyrus ussuriensis Maxim. in China
Source: PLoS One. 2015 Aug 7;10(8):e0133686. doi: 10.1371/journal.pone.0133686 (PMC4529180; doi:10.1371/journal.pone.0133686)
Supplement: S5 Table — (DOCX) [file pone.0133686.s006.docx]

S5 Table. Pairwise genetic differentiation (*F_ST_*) among the prior populations using cpSSRs

|  | IMQS | IMTHL | IMPJG | IMRSL | IMLMD | IMSLG | HLYCS | HLFYX | HLSWX | HLYCS2 | HLYCS1 | JLGZL | HLMTZ |
| --- | --- | --- | --- | --- | --- | --- | --- | --- | --- | --- | --- | --- | --- |
| IMQS | 0.000 | - | - | - | - | - | ** | ** | ** | ** | ** | ** | ** |
| IMTHL | 0.000 | 0.000 | - | - | - | - | ** | ** | ** | ** | ** | ** | ** |
| IMPJG | 0.000 | 0.000 | 0.000 | - | - | - | ** | ** | ** | ** | ** | ** | ** |
| IMRSL | 0.192 | 0.266 | 0.204 | 0.000 | - | - | ** | ** | ** | ** | ** | ** | ** |
| IMLMD | 0.189 | 0.248 | 0.136 | 0.067 | 0.000 | - | ** | ** | ** | ** | ** | ** | ** |
| IMSLG | 0.000 | 0.000 | 0.000 | -0.015 | 0.124 | 0.000 | ** | ** | ** | ** | ** | ** | ** |
| HLYCS | 0.976 | 0.981 | 0.977 | 0.939 | 0.955 | 0.967 | 0.000 | - | - | ** | ** | ** | ** |
| HLFYX | 0.990 | 0.992 | 0.990 | 0.964 | 0.973 | 0.986 | 0.020 | 0.000 | - | ** | ** | ** | ** |
| HLSWX | 0.965 | 0.973 | 0.967 | 0.908 | 0.935 | 0.951 | -0.017 | -0.030 | 0.000 | - | ** | * | ** |
| HLYCS2 | 0.867 | 0.894 | 0.872 | 0.749 | 0.808 | 0.824 | 0.205 | 0.202 | 0.062 | 0.000 | ** | ** | ** |
| HLYCS1 | 0.920 | 0.940 | 0.924 | 0.793 | 0.860 | 0.885 | 0.832 | 0.860 | 0.763 | 0.609 | 0.000 | - | * |
| JLGZL | 0.689 | 0.742 | 0.698 | 0.515 | 0.601 | 0.615 | 0.395 | 0.413 | 0.305 | 0.226 | 0.100 | 0.000 | * |
| HLMTZ | 0.958 | 0.967 | 0.960 | 0.901 | 0.927 | 0.942 | 0.704 | 0.755 | 0.608 | 0.483 | 0.818 | 0.421 | 0.000 |
