# Supplementary material for: Population Structure of and Conservation Strategies for Wild Pyrus ussuriensis Maxim. in China
Source: PLoS One. 2015 Aug 7;10(8):e0133686. doi: 10.1371/journal.pone.0133686 (PMC4529180; doi:10.1371/journal.pone.0133686)
Supplement: S6 Table — (DOCX) [file pone.0133686.s007.docx]

S6 Table. Analysis of molecular variance (AMOVA) averaged across 20 nSSR using 275 individuals of Iwateyamanashi (*P. ussuriensis* Maxim. var *aromatica*) from north east Japan

| Source of variation | Percentage of variation |
| --- | --- |
| Among populations | 7.20% |
| Within populations | 91.14% |
