# Supplementary material for: Population Structure of and Conservation Strategies for Wild Pyrus ussuriensis Maxim. in China
Source: PLoS One. 2015 Aug 7;10(8):e0133686. doi: 10.1371/journal.pone.0133686 (PMC4529180; doi:10.1371/journal.pone.0133686)
Supplement: S7 Table — (DOCX) [file pone.0133686.s008.docx]

S7 Table. The characteristic of floral morphologies for wild *P. ussuriensis* Maxim in Inner Mongolia, Heilongjiang, and Ussurian pear cultivars

| Population | Number of individual | Flower diameter (mm)* | Petal length (mm)* | Petal witdth (mm)* | Petal length / Petal width* | Peduncle length (mm)* |
| --- | --- | --- | --- | --- | --- | --- |
| IMQS | 10 | 28.30±5.81 | 12.90±2.79 | 7.85±1.28 | 1.68±0.23 | 7.90±2.64 |
| IMTHL | 6 | 26.72±6.27 | 13.37±6.27 | 7.97±2.15 | 1.71±0.38 | 6.10±2.00 |
| IMPJG | 8 | 23.44±4.16 | 11.56±2.88 | 6.35±1.53 | 1.85±0.33 | 7.19±3.66 |
| IMLMD | 3 | 25.59±6.28 | 10.49±2.51 | 8.45±1.45 | 1.41±0.17 | 7.43±1.81 |
| IMSLG | 7 | 21.38±3.70 | 10.62±3.70 | 5.95±1.32 | 1.83±0.34 | 5.40±1.79 |
| HLYCS3 | 10 | 30.81±3.95 | 15.31±1.80 | 10.62±1.13 | 1.58±0.26 | 11.38±2.68 |
| HLFYX | 10 | 41.79±3.50 | 19.76±1.88 | 12.66±1.43 | 1.58±0.19 | 14.05±2.65 |
| HLMTZ | 10 | 43.14±2.83 | 20.27±1.53 | 14.28±1.45 | 1.44±0.10 | 25.09±4.53 |
| Cultivars | 26 | 36.75±4.95 | 16.81±2.59 | 14.41±3.02 | 1.19±0.19 | 33.93±7.58 |
| Average** | 11.22 | 30.81±7.80 | 14.04±4.64 | 9.86±3.31 | 1.59±0.21 | 12.70±9.35 |

*: Mean value ± SD, **: Average of 8 populations.
