# Supplementary material for: Population Structure of and Conservation Strategies for Wild Pyrus ussuriensis Maxim. in China
Source: PLoS One. 2015 Aug 7;10(8):e0133686. doi: 10.1371/journal.pone.0133686 (PMC4529180; doi:10.1371/journal.pone.0133686)
Supplement: S8 Table — (DOCX) [file pone.0133686.s009.docx]

S8 Table. The characteristics of fruit morphologies for wild *P. ussuriensis* Maxim. in Inner Mongolia, Heilongjiang, and an Ussurian pear cultivar ‘Nanguoli’

| Population | Number of individual | Fruit length (mm)* | Fruit witdth (mm)* | Fruit length / Petal width* | Peduncle length (mm)* | Calyx presistence |
| --- | --- | --- | --- | --- | --- | --- |
| IMQS | 19 | 28.93±2.96 | 31.43±3.26 | 0.92±0.04 | 13.51±3.59 | 0.44±0.27 |
| IMTHL | 13 | 30.28±2.46 | 32.74±2.39 | 0.92±0.03 | 11.66±3.05 | 0.59±0.22 |
| IMPJG | 11 | 26.42±1.67 | 28.18±2.25 | 0.94±0.03 | 11.64±1.80 | 0.30±0.23 |
| IMSLG | 10 | 25.41±2.13 | 28.08±1.59 | 0.90±0.04 | 12.15±2.22 | 0.43±0.30 |
| HLYCS3 | 11 | 27.03±2.58 | 31.15±2.58 | 0.87±0.04 | 15.23±2.37 | 0.30±0.19 |
| Nanguoli (Cultivar) | 1 | 52.12 | 53.45 | 0.98 | 25.38 |  |
|  |  |  |  |  |  |  |
| Average** | 10.8 | 27.61±1.97 | 30.32±2.08 | 0.91±0.03 | 12.84±1.54 | 0.41±0.12 |

*: Mean value ± SD, **: Average of 5 populations.
