# Supplementary material for: Population Structure of and Conservation Strategies for Wild Pyrus ussuriensis Maxim. in China
Source: PLoS One. 2015 Aug 7;10(8):e0133686. doi: 10.1371/journal.pone.0133686 (PMC4529180; doi:10.1371/journal.pone.0133686)
Supplement: S9 Table — (DOCX) [file pone.0133686.s010.docx]

S9 Table. Allelic richness with and without HLYCS1 in *P. ussuriensis*

Ar (Original) Ar (recalculated 12 alleles )

IMQS 1.46 1.71

IMTHL 1.48 1.59

IMPJG 1.47 1.68

IMRSL 1.45 1.63

IMLMD 1.46 1.72

IMSLG 1.46 1.63

HLYCS1 1.55 1.76

HLYCS2 1.39 1.85

HLYCS3 1.46 1.77

HLFYX 1.63 1.60

HLSWX 1.52 1.83

HLMTZ 1.44 1.63

JLGZL 1.61 1.82

SL 1.72 1.85

XJL 1.73 1.82

BL 1.79 1.82

QZL 1.76 1.83

EUR 1.73 1.81

IWT 1.71 1.72

JAP 1.67 1.67
